# P-739. Missed Asymptomatic Neurosyphilis Despite Fourfold Reduction in RPR Among People Living with HIV: A Seven-Year Single-Center Analysis

**DOI:** 10.1093/ofid/ofaf695.950

**Published:** 2026-01-11

**Authors:** Samadhi Patamatamkul, Kanphai Wongjarit

**Affiliations:** Department of Medicine, Faculty of Medicine, Mahasarakham University, Kantharawichai, Maha Sarakham, Thailand; Chulalongkorn University, Bangkok, Krung Thep, Thailand

## Abstract

**Background:**

CSF analysis for asymptomatic latent syphilis in people living with HIV (PLWH) is indicated under specific conditions. This study explores asymptomatic neurosyphilis (ASN) in PLWH, particularly those with an incomplete serological response (ISR) after adequate treatment.

**Methods:**

A retrospective study was conducted from 2017 to 2024 at a tertiary care facility associated with Mahasarakham University. We included patients aged ≥ 18 years who tested positive for treponemal antibodies (TPHA) and had completed at least 24 months of follow-up with serial serum RPR tests. Participants who underwent CSF analysis were reviewed. ISR is defined as an RPR titer of ≥ 1:2 after 24 months of adequate treatment, despite a fourfold reduction in titer, and without signs of reinfection.

**Results:**

Among 185 PLWH and positive TPHA, 42.2% (78/185) completed 24 months of follow-up. There was a total of 17.9% (14/78) having an incomplete serological response (ISR). There were 14 CSF analyses performed among 13 individuals, of which 9/14 (64.3%) had no indications recommended by the CDC 2021 guidelines. Of these, 33.3% were symptomatic (2 with ocular diseases and 1 with tinnitus), while the remainder were asymptomatic. Among the asymptomatic group, 4/6 had an ISR with initial RPR values ≥ 64 [(256,32), (128,16), (256,32), (64,8)]; 1/6 had an ISR(16,4), and one was part of a work-up for cryptococcal antigenemia. Neurosyphilis was diagnosed in 2/9 (22.2%): syphilitic uveitis and ASN. Notably, 20% (1/5) of those with ISR who underwent CSF examinations were diagnosed with ASN. This virologically suppressed case (CD4 counts of 723 cells/mm^3^, 26%) showed a reduction in RPR titer from 128 to 16 and was confirmed to have ASN by positive CSF TPHA and FTA-ABS IgG, along with a CSF WBC count of 14 cells/mm³ and a protein level of 63 mg/dL. No significant differences in baseline characteristics were found between ISR participants with or without ASN.

**Conclusion:**

A high rate (25%, 1/5) of possible ASN was observed among participants with ISR after treatment for early syphilis. Despite the small sample size, this study underscores significant management implications for this high-risk group, which exhibited a higher risk for ASN in a setting with limited data.

This research project was financially supported by Mahasarakham University.

**Disclosures:**

All Authors: No reported disclosures

